# Can a Public and Patient Involvement (PPI) Informed Participant Information Leaflet (PIL) Improve Trial Recruitment, Retention, and Quality of Decision Making? Results of a Randomised Controlled Double‐Blind Study Within a Trial (SWAT)

**DOI:** 10.1111/hex.70321

**Published:** 2025-06-08

**Authors:** Linda O'Neill, Peter Knapp, Suzanne L. Doyle, Sanela Begic, Emily Smyth, Neil Kearney, Sophie Grehan, Adwoa Parker, Peter Browne, Ricardo Segurado, Deirdre Connolly, Jacintha O'Sullivan, John V. Reynolds, Emer Guinan, Juliette Hussey

**Affiliations:** ^1^ Discipline of Physiotherapy, School of Medicine, Trinity College Dublin the University of Dublin Dublin Ireland; ^2^ Trinity St James's Cancer Institute Dublin Ireland; ^3^ Clinical Research Centre, School of Medicine University College Dublin Dublin Ireland; ^4^ Hull York Medical School and the Department of Health Sciences University of York York UK; ^5^ School of Biological and Health Sciences Technological University Dublin Dublin Ireland; ^6^ Department of Surgery School of Medicine Trinity College Dublin and St James's Hospital Dublin Ireland; ^7^ York Trials Unit, Department of Health Sciences University of York York UK; ^8^ School of Public Health, Physiotherapy and Sport Sciences University College Dublin Dublin Ireland; ^9^ Discipline of Occupational Therapy, School of Medicine, Trinity College Dublin the University of Dublin Dublin Ireland

**Keywords:** participant information, public and patient involvement, recruitment, retention, trial understanding

## Abstract

**Introduction:**

Public and patient involvement (PPI) may be utilised to improve aspects of trial conduct. This study within a trial (SWAT) aimed to examine if a PPI‐informed participant information leaflet (PIL) could improve recruitment, retention, and quality of decision‐making of an exercise‐based rehabilitation trial (RESTORE II) for upper gastrointestinal (UGI) cancer survivors.

**Methods:**

This SWAT was conducted over two phases. Phase 1 applied qualitative methods to develop and refine a PPI‐informed PIL. Phase 2 embedded a randomised controlled double‐blind SWAT within the RESTORE II trial, comparing a standard PIL (PIL A) to the PPI‐informed PIL (PIL B) in terms of recruitment, retention, and quality of decision‐making (Decision Making Questionnaire [DMQ]).

**Results:**

Phase 1 recruited 16 PPI members (mean age 67.01 [9.28] years, mostly male [81.25%] and all UGI cancer survivors). Participants reviewed the standard PIL A and made suggestions for improvement, including revisions to the layout, title, text, provision of key information on the first page, and greater emphasis on the potential benefits of participation. This feedback was used to draft the alternative PIL B, which the Phase 1 participants reviewed and refined through minor changes to the appearance, text, and layout. In Phase 2, 307 potential RESTORE II trial participants were randomised to receive either PIL A (*n* = 154) or PIL B (*n* = 153). The overall recruitment rate was 28.7%. (PIL A 26.6% vs. PIL B 30.7%, OR 1.22 [95% CI 0.74 to 2.01, *p* = 0.43]), retention was 84.1% (PIL A 85.4% vs. PIL B 83.0%, OR 0.84 [95% CI 0.26 to 2.65, *p* = 0.760]). No significant difference in mean (SD) DMQ scores was observed: PIL A: 29.1 (4.4) vs. PIL B: 29.1 (5.1) (mean difference 0.03, 95% CI −1.64 to 1.69, *p* = 0.49).

**Conclusions:**

A PPI‐informed PIL did not improve recruitment, retention, or decision‐making for the RESTORE II trial.

**Patient or Public Contribution:**

Patients with UGI cancer informed the development of the interventional PIL B. Author P.B. provided input from the patient's perspective throughout the SWAT as a member of the Trial Steering Committee, providing oversight to the SWAT management, and contributed to analysis and the production of this manuscript.

**Trial Registration:**

SWAT Registration: The Northern Ireland Hub for Trials Methodology Research, SWAT Store, SWAT 100 https://www.qub.ac.uk/sites/TheNorthernIrelandNetworkforTrialsMethodologyResearch/FileStore/Filetoupload,914713,en.pdf. Host Trial Registration Clinical Trials. gov https://clinicaltrials.gov/study/NCT03958019.

## 1 Introduction

Advancements in cancer detection and treatments have led to a growing number of cancer survivors globally [1]. Accordingly, there has been a surge in research to investigate strategies aiming to optimise quality of life in cancer survivorship and mitigate any negative sequelae of cancer and its treatment [2]. Exercise‐based rehabilitation is an example of such a strategy which has increasingly demonstrated effectiveness, with strong evidence that three times weekly moderate‐intensity aerobic training and twice weekly resistance training may alleviate symptoms of anxiety, depression, fatigue and improve quality of life and physical functioning for cancer survivors [3]. Nevertheless, questions remain regarding its impact on other cancer‐related symptoms, optimum dosing and its effects in cancers other than breast, prostate, and colorectal cancer [3, 4]. To this end, trialists in cancer survivorship are continuing to explore the impact of exercise‐based rehabilitation in cancer survivorship [3, 5]. However, recruitment and retention in these types of trials may be problematic, limiting the generalisability and impact of findings and ultimately delaying the integration of much‐needed rehabilitation services into survivorship care [6, 7]. Consequently, it is incumbent upon cancer survivorship trialists to explore strategies that may ameliorate recruitment and retention rates.

Increasingly, there has been a growth in trials methodology research, which is the study of how we conduct trials and is critical to developing and enhancing their conduct [8]. Trial methodologists in the United Kingdom and Ireland have worked collaboratively over the past decade to establish key priorities for trials methodology research [9]. Consistently, strategies to improve recruitment and retention have been identified by trialists as priorities for investigation [8], with public and patient involvement (PPI) being one such intervention meriting exploration [10]. PPI refers to the active involvement of the public and patients in research [11] and is now an integral part of the research process. Not only is PPI essential to ensuring the patient's voice is to the fore of clinical research, but increasingly, evidence supports that PPI may positively impact the conduct of clinical trials [10, 12]. For example, a systematic review and meta‐analysis by Crocker et al. investigated the impact of PPI interventions on clinical trial recruitment and retention rates. PPI interventions included in the review were: involving patients or lay people in the design of recruitment and retention strategies (*n* = 8), involving patients or lay people in developing patient‐facing information (*n* = 12) and involving patients or lay people in directly recruiting and retaining participants (*n* = 10). The most robust evidence was found in support of the use of PPI in developing patient‐facing information, for which evidence from seven randomised studies was available, and meta‐analysis determined that such PPI interventions modestly improved the odds of participant recruitment (odds ratio 1.16, 95% confidence interval [CI] 1.01 to 1.34). Consequently, given the need to enhance recruitment to rehabilitative trials in cancer survivorship, we sought to explore whether a PPI‐informed participant information leaflet (PIL) could positively impact recruitment in an exercise‐based rehabilitation trial in cancer survivorship.

The RESTORE II trial was a two‐armed, single blind, randomised controlled trial comparing a multidisciplinary programme of supervised and home‐based aerobic and resistance exercise, one‐to‐one dietary counselling and group‐based multidisciplinary education to standard care in upper gastrointestinal (UGI) cancer survivorship [13]. A pilot randomised controlled trial of this intervention achieved positive recruitment and retention rates of 40.4% and 93.0%, respectively [14]. Given the potential benefits of PPI‐informed participant information, investigators leading the RESTORE II trial developed a study within a trial (SWAT) with a view to enhancing trial recruitment and retention further. The overall aim of the SWAT was to explore the impact of the PPI‐informed PIL in the RESTORE II trial. Specific objectives were to: (i) convene a panel of PPI members (UGI cancer survivors and/or family members) to develop and refine a PPI‐informed PIL and (ii) examine the impact of the PPI‐informed PIL on trial recruitment rates, trial retention, and quality of decision‐making about the trial. This manuscript is presented in line with the SWAT reporting guidelines [15].

## 2 Methods

### 2.1 Study Design

**Figure 1:**
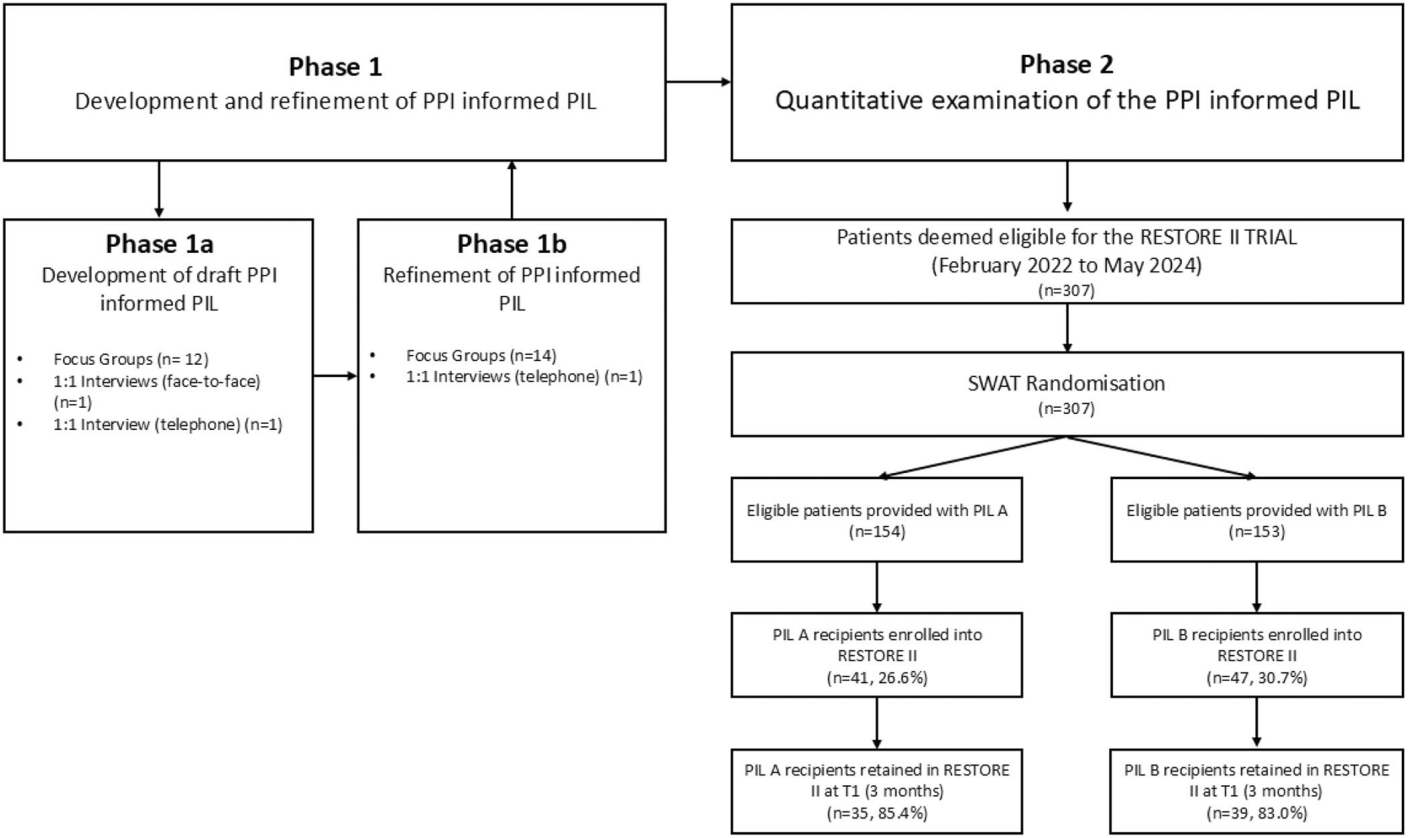
Flow of participants through the study.

The study design has been described in detail elsewhere [16]. Research ethics approval for the SWAT and host trial was obtained. In brief, the study was conducted over two distinct phases: Phase 1 (development) and Phase 2 (evaluation) (Figure 1). In Phase 1, PPI representatives informed the development of the interventional PIL. In Phase 2, a randomised controlled SWAT was hosted within the RESTORE II trial at St James's Hospital Dublin (SJH), Ireland, a national centre for UGI cancer care, to compare the PPI‐informed PIL to the standard PIL.

### 2.2 Recruitment

Phase 1 recruited PPI members, namely patients and/or their family members who had previously undergone treatment for cancer of the oesophagus, stomach, pancreas or liver. Exclusion criteria included communication or cognitive difficulties, which would inhibit the ability to take part in the interview/focus group process. A three‐pronged approach to PPI recruitment was implemented. First, participants of the RESTORE I trial or feasibility study [14, 17] were invited by letter to participate; second, PILs were provided to patients attending the UGI cancer clinic at SJH during October 2019; and finally individuals could self‐refer by responding to adverts disseminated by the host trial charity partners, the Irish Cancer Society and the Oesophageal Cancer Fund. Phase 1 participants were required to give written informed consent. They received a small gratuity voucher (€20) for their participation, and parking costs were covered at SJH for those attending in person.

Phase 2 of the SWAT included all potential RESTORE II trial participants. The RESTORE II Trial [13] recruited participants with UGI cancer from SJH who had completed treatment with curative intent, were greater than 3 months post‐surgery and were free from significant co‐morbidity that would preclude maximal exercise testing.

### 2.3 Phase 1—Development and Refinement of PPI‐Informed PIL

Phase 1 was conducted over two distinct subphases: Phase 1a (development of the PPI‐informed PIL) and Phase 1b (refinement of the PPI‐informed PIL).

#### 2.3.1 Phase 1a—Development of the PPI‐Informed PIL

In Phase 1a, patients or close family members were invited to take part in a one‐to‐one semi‐structured interview or a focus group discussion. All focus groups and face‐to‐face interviews were conducted in the Clinical Research Facility (CRF) at SJH. Participants who were unable to attend SJH at the time of the focus groups/interviews were given the opportunity to complete a 1:1 interview remotely via video or telephone call.

The focus groups and interviews were led by Professor Knapp, a male researcher experienced in PPI engagement and qualitative research and who had no other involvement in the RESTORE II trial. The project manager, Dr O'Neill, a female researcher experienced in cancer rehabilitation, was present for all interviews and focus groups to provide expertise on the host trial. Focus groups and interviews followed a topic guide agreed by the research team, which asked participants to comment on the standard RESTORE II Trial PIL (PIL A). PIL A was developed before the first focus group by the research team according to the institutional template. Participants were invited to make suggestions on how to enhance the content, language, structure and visual appeal of PIL A. The Consensus‐Orientated‐Decision‐Making model [18] was used to guide the focus group participants to consensus. Following the completion of the Phase 1a focus groups/interviews, the recordings were transcribed verbatim. A thematic analysis was performed guided by the Braun and Clarke model [19] by Professor Knapp and Dr O'Neill. The analysis had both inductive and deductive aspects and involved a period of data familiarisation (reading and re‐reading of transcripts), line‐by‐line coding to identify repeating ideas, followed by the grouping of codes into themes and sub‐themes that were framed as actionable recommendations. These key recommendations were then applied to develop an initial draft of the PPI‐informed PIL in consultation with a graphic design company (Making Sense Design Ltd.).

#### 2.3.2 Phase 1b—Refinement of the PPI‐Informed PIL

Two months after the completion of Phase 1a, participants were asked to return for a second focus group/interview. Individuals who had expressed an interest in participation but were unable to attend for Phase 1a were also eligible to take part. Participants were provided with the draft PPI‐informed PIL via post a week before the Phase 1b focus group/interview. In Phase 1b, participants critiqued the PPI‐informed PIL that had been drafted based on the Phase 1a findings and focused specifically on its structure, content, language, and visual appeal. As per Phase 1a, focus groups/interviews were audio recorded, transcribed verbatim and analysed using the thematic approach described in Phase 1a by Professor Knapp and Dr O'Neill. The PIL was then refined by the graphic design company. The final draft of the PPI‐informed PIL was then circulated to Phase 1 participants for approval before submission for ethical approval for use in the RESTORE II trial. The finalised PIL was printed professionally, in colour, and A4 booklet format.

### 2.4 Phase 2—Evaluation

Following ethical approval, the resultant PPI‐informed PIL was tested in a prospective, randomised, double blind, parallel trial design. Potential RESTORE II trial participants were randomised to receive either the standard PIL (PIL A) or the PPI informed PIL (PIL B) (both available at https://doi.org/10.17605/OSF.IO/WH3YM) [20]. Recruitment to RESTORE II was completed via the institutional database at SJH. Following medical team approval, eligible trial participants received a letter from the lead clinical investigator Professor Reynolds and the project manager Dr O'Neill inviting them to express an interest in participating in the RESTORE II trial along with PIL A or PIL B (as per SWAT allocation), the Decision‐Making Questionnaire (DMQ), and a stamped addressed envelope.

#### 2.4.1 Randomisation

Randomisation was overseen by the CRF at SJH. Participants were randomised in a 1:1 ratio, using block randomisation (permuted blocks of 2, 4, 6, 8), by an online randomisation system (www.sealedenvelope.com) and were shared with and accessed by members of the research team not directly involved in recruitment.

#### 2.4.2 Blinding

Participants invited to participate in the RESTORE II trial were blinded to the SWAT, that is they received one version of the PIL and were not informed that other people were receiving a different PIL. Trial members responsible for recruitment were also blinded to the SWAT allocation.

#### 2.4.3 Outcomes

The primary outcome for this SWAT was the recruitment rate to the RESTORE II trial, defined as ‘the proportion of eligible participants who agreed to take part in the RESTORE II trial’. Secondary outcomes were retention and quality of decision‐making. Retention was defined as ‘the proportion of randomised participants who participated in the RESTORE II host trial up to and including the first follow‐up data collection time point (T1—post‐intervention assessment completed 12 weeks post‐enrolment)’.

Quality of decision‐making was measured using a modified version of the TRECA DMQ [21], which evaluates the utility of information to help make informed decisions about trial participation. The DMQ consists of nine Likert scale questions, which are scored from 0 to 4 and have a total possible score range of 0–36, with a higher score indicating better quality of decision‐making [22]. In the case of partial non‐completion of the measure, up to three missing responses were permitted, and the total score was calculated by replacing the missing values with the participant's mean value of the completed items [22]. There were also three ‘open text’ questions which gave respondents the opportunity to highlight parts of the PIL that were explained well, items that may require further information, and to make any additional comments [21]. Responses to these items were categorised using content analysis. The DMQ was provided to potential RESTORE II trial participants along with the recruitment letter and allocated PIL. Potential RESTORE II trial participants received a follow‐up phone call from the recruitment team to check their interest in participation 1 week after they received the trial information. During that call, researchers asked potential RESTORE II trial participants to complete the DMQ and return it in the stamped, addressed envelope provided.

#### 2.4.4 Sample Size

The sample size for the Phase 2 evaluation was determinant on recruitment to the RESTORE II host trial. A sample size of approximately 300 patients was anticipated based on the accrual rate achieved in the RESTORE pilot RCT (40%) [14, 16].

### 2.5 Statistical Analysis

Statistical analyses were performed using SPSS 29 (SPSS Inc., Chicago, Illinois, the United States), following intention‐to‐treat principles. Baseline characteristics of Phase 1 SWAT and RESTORE II participants were analysed descriptively. Continuous data was presented as mean (standard deviation), and categorical data as counts and percentages. For analysis of recruitment and retention rates, logistic regression was used to determine odds ratios and their associated 95% CIs and *p* values. Independent *t*‐tests were used to determine differences between groups (PIL A and PIL B) for the total DMQ score.

## 3 Results

### 3.1 Phase 1 PPI Member Characteristics

Phase 1 recruited 16 PPI members (Table 1). Twelve had previously completed the RESTORE Programme (RESTORE Feasibility Study or Pilot RCT) [3, 4], three were recruited from the UGI Cancer Clinic at SJH and one responded to adverts on social media.

**Table 1:** Phase 1 participant characteristics.

Participant ID	Age	Gender	Ethnicity	English first language	Employment status	Highest level of education completed	Cancer type	Time since diagnosis (years)	History of rehabilitation trial participation	Phase 1a	Phase 1b
PIL01	50	Male	White	Yes	Long term sick leave	Third level	Oesophagus	1	No	Phone interview	N/A
PIL02	73	Male	White	Yes	Retired	Third level	Oesophagus	6	Yes	Focus group	Focus group
PIL03	70	Male	White	Yes	Retired	Primary	Oesophagus	6	Yes	Focus group	Focus group
PIL04	67	Male	White	Yes	Retired	Upper secondary	Stomach	5	Yes	Focus group	Phone interview
PIL05	77	Male	White	Yes	Retired	Upper secondary	Oesophagus	5.5	Yes	Focus group	Focus group
PIL06	57	Female	White	Yes	Employed (FT)	Third level	Oesophagus	9	Yes	Focus group	Focus group
PIL07	66	Male	White	Yes	Retired	Third level	Oesophagus	4	Yes	Focus group	Focus group
PIL08	60	Male	White	Yes	Retired	Primary	Pancreas	2.25	No	Face‐to‐face interview	Focus group
PIL09	48	Female	White	Yes	Long term sick leave	Third level	Oesophagus	1	Yes	Focus group	Focus group
PIL10	59	Female	White	Yes	Retired	Third level	Oesophagus	6	Yes	Focus group	Focus group
PIL11	67	Male	White	Yes	Retired	Lower secondary	Oesophagus	1	No	Focus group	Focus group
PIL12	61	Male	White	Yes	Employed (PT)	Third level	Oesophagus	3.5	Yes	Focus group	Focus group
PIL13	74	Male	White	Yes	Retired	Lower secondary	Oesophagus	5	Yes	Focus group	Focus group
PIL14	77	Male	White	Yes	Retired	Primary	Oesophagus	7	Yes	Focus group	Focus group
PIL15	77	Male	White	Yes	Retired	Upper secondary	Oesophagus	4	Yes	N/A	Focus group
PIL16	61	Male	White	Yes	Employed (PT)	Upper secondary	Oesophagus	5	Yes	N/A	Focus group

Abbreviations: FT = full time, PT = part time, Lower Secondary = irish junior certificate, Upper Secondary = irish leaving certificate.

PPI members were mostly male (*n* = 13, 81.25%), retired (*n* = 11, 68.75%) and all white with English as their first language. The highest level of education completed was reported as follows: third level (*n* = 7, 43.75%), upper secondary (*n* = 4, 25%), lower secondary (*n* = 2, 12.5%) and primary (*n* = 3, 18.75%). All had a history of cancer diagnosis; oesophageal (*n* = 14, 87.5%), stomach (*n* = 1, 6.25%) and pancreas (*n* = 1, 6.25%), and were a mean (SD) 4.4 (2.3) years post‐diagnosis. Thirteen had previously participated in a rehabilitation trial (81.2%). Fourteen took part in Phase 1a, with twelve taking part in a focus group (85.7%), one completed a telephone interview (7.1%) and one took part in a face‐to‐face interview (7.1%). Fifteen took part in Phase 1b, with 14 taking part in a focus group (93.3%) and 1 taking part in a telephone interview (6.7%).

### 3.2 Phase 1 Key Findings

Phase 1a focus group and interviews resulted in five key recommendations; (i) revise how the PIL is presented, (ii) revise how the trial title is presented, (iii) provide all key information on the first page, (iv) emphasise the potential benefits of trial participation and (v) revise text and diagrams to improve readability. In Phase 1b, specific revisions on the visual appearance, text and layout were recommended. Specific details of all Phase 1 recommendations and supporting quotes are presented in Table 2.

**Table 2:** Recommendations made during Phase 1 Focus Groups/Interviews.

	Recommendation	Specifics	Supporting quotes
**Phase 1a**			
1	Revise how PIL is presented	Participants recommended key changes to how the PIL was presented to improve its visual appeal, including: Assign numbers to sections to improve navigability The use of colour to enhance the visual appeal	PIL07: *I would say that, that in one way its fine but its functional, it certainly does all it needs to do but in terms of layout, it might be possible to just make it more attractive on the eye!* PIL02: *See the way you have one with the numbers, possibly on our one here we have a number whatever it is and if you have the whole list at the top starting off* PIL08: *Yes the highlighting if you wanted to emphasise some items more the little bit of colour scheme is catchy it catches the eye and what not*.
2	Revise how the trial title is presented	Participants recommended enhancing the readability of the trial title by: Changing the title on PIL to put the trial name ‘RESTORE II’ first and then the full title Keeping trial letters in the same font style, for example, RESTORE II not ReStOre II	PIL06: *I suppose again I have no issue reading anything but if I received this in the post and I struggled some what I would run a mile from reading ‘Rehabilitation Strategies following Oesophagogastric and blllllllleblaaaaa whatever!!’ So that's the first thing that hit me, that it's a bit of a belter of a headline* PIL07: *But I think it took me a while to work that out, I actually just would take the capital letters out or else put them all in caps for ReStOre, it's like O'Toole … you're looking what's a big S and you don't know if it's some sort of mathematical formula or what … but I just think it would be lost on some*
3	Provide all key information on first page	Participants recommended presenting key information regarding the trial on the first page. The following information should be included: A list of contents Contact information for key study personnel A summary of key points on the trial	PIL11: *You've got to sell yourself first, and you sell yourself on the first page* PIL09: *I quite like that you've got a contents so you can quickly find what is important to you*. PIL01: *Perhaps a contact number if there's any part of this document that you don't understand that someone can call*. PIL07: *But I would just say a one frontal summary page and then it's all there for those who want to get into the detail, but it must be possible to summarise that in one page*
4	Emphasise the potential benefits of trial participation	Participants recommended clearly identifying the benefits of participation. Specifically, participants recommended emphasising the: Potential physical and psychosocial benefits of participation Potential benefits that participation may have for others	PIL09: *I suppose it's important to mention that people who have done these type of studies before us have helped to improve care and treatment…. Yeah it's worth mentioning that this has happened before and it has been useful* PIL08: *Well the personal benefit is always important, because everyone I think, if you chose anybody on this earth you as a person will always come first, you know?…. If I was healthy enough or lucky enough to be able to help somebody else I always had a desire to be able to reach out and help someone else and this is almost kind of similar in so far as its close to me going to my final destination it may be my last opportunity to reach out and help someone else so I want to be involved in that too, but number one it is a personal interest*
5	Revise text and diagrams to improve readability	Participants recommended revising the text and diagrams to improve readability. Key recommendations included: Use of simpler language and shorter paragraphs Remove duplication Make diagrams clearer Reduce extensiveness of the data protection section	PIL07: *I mean just one thing you could probably paragraph it a bit better, it's just a bit dense, the layout is very dense*. PIL09: *Yeah there are some duplication of the information. Like on page 3, physical performance, you've got that the test will be performed on a stationary bike and then this will require you to exercise on a stationary bike until exhaustion. So it's basically the same information, so there's just little bits* *PIL11: You're showing me a drawing [referring to flow diagram of trial] of that, that you have to try and figure out. The drawing is not really good*. PIL03: *Half of the document is to do with this GPDR or whatever it is, and I totally have a bee in my bonnet I think that's gone over the top*
**Phase 1b**			
1	Revise the visual presentation of the PIL	Participants recommended visual changes to improve the presentation. Specific points raised included: Font size in graphics on pages 2 and 3 is quite small Light blue shading on graphics and Page 1 is a little dark Need to darken the font throughout Changes to layout, specifically moving the ‘feedback’ subsection from Section 3 to Section 4.	PIL02: *Increase the size of it, yeah. (referring to font size in graphics page 2 and 3)* PIL04: *Yeah the colours are grand, yeah. Maybe the lighter blue is just a little bit hard to* PIL15: *I don't think you need to enlarge it, you just need to make it darker, more bold, you know?* PIL07: *This thing here about the feedback etcetera, is more closely allied what is happening after you've done it*.
2	Revise text content to enhance readability	Participants recommended changes to text content to enhance readability and understanding. Specifically, participants recommended revision of some terminology to language patients familiar/comfortable with	PIL07: *Just one thing, I don't know whether you're talking about the language, but I found the use of the word ‘food pipe’ is odd, like people who are doing this, they know what the oesophagus is, and they know what oesophageal cancer is, I should imagine. And the other things are, even putting in non‐medical terms, I'd be tempted just to use the word oesophagus. Everyone knows what it is, the use of the word ‘food pipe’ almost suggests that people need, it's kind of condescending to be saying that. It's like simplifying it. You're saying ‘look, you don't know what the oesophagus is, so use the term food pipe’. I've never called it the ‘food pipe’ in my life, I don't think many people do*. PIL12: *There's a sentence at the bottom of page two, just where you see the diagram. It's about the control group. ‘The control group will continue with their usual hospital care’. Now when I look at that, I say ‘well, thankfully I'm not getting any hospital care’, and I gather that's the same with a number of people around the table. It just seems a bit presumptive that people are getting care? I mean, many people are just back living their lives again*.

### 3.3 Phase 2 Participants

The RESTORE II SWAT commenced in February 2022. A total of 307 participants met the RESTORE II eligibility criteria from February 2022 to May 2024. One hundred and fifty‐four were randomised to receive PIL A and 153 were randomised to receive PIL B (Figure 1). Baseline characteristics are only available for participants who consented to the RESTORE II trial due to data protection legislation (Table 3). The mean (SD) age was 67.0 (9.3), participants were mostly male (*n* = 60, 68.2%), all were ethnically white, and for all but one participant, English was their first language (98.9%).

**Table 3:** Demographic characteristics of SWAT participants who consented to participation in RESTORE II.

		PIL A (*n* = 41)	PIL B (*n* = 47)	Overall (*n* = 88)
Age in years, mean (SD)		67.3 (9.7)	66.8 (8.9)	67.0 (9.3)
Gender, *n* (%)				
Male		31 (75.6)	29 (61.7)	60 (68.2)
Female		10 (24.4)	18 (38.3)	28 (31.8)
Ethnicity, *n* (%)				
White		41 (100)	47 (100)	88 (100)
English as first language, *n* (%)		41 (100)	46 (97.9)	87 (98.9)
Cancer Type, *n* (%)				
Oesophageal		31 (75.6)	25 (53.2)	56 (63.6)
Gastric		10 (24.4)	22 (46.8)	32 (36.4)
Time since diagnosis (months), median (range)		52 (7 to 136)	25 (3.5 to 145)	33 (3.5 to 145)
Cancer treatment				
Surgery		41 (100)	47 (100)	88 (100)
Neoadjuvant treatment		26 (63.4)	34 (72.3)	60 (68.18)
Adjuvant treatment		14 (34.1)	20 (42.6)	34 (38.63)
RESTORE II trial allocation				
Control		27 (65.9)	18 (38.3)	45 (51.1)
Intervention		14 (34.1)	29 (61.7)	43 (48.9)

### 3.4 Phase 2 Results

#### 3.4.1 Primary Analysis

##### 3.4.1.1 Recruitment

Of the 307 participants approached to take part in the RESTORE II trial, 88 consented to participation, representing a recruitment rate of 28.7%. For those receiving the standard PIL A, the recruitment rate was 26.6% (*n* = 41/154). For those receiving the PPI‐informed PIL B, the recruitment rate was higher, 30.7% (*n* = 47/153). Logistic regression gave an OR of 1.22 (95% CI 0.74 to 2.01, *p* = 0.43), meaning that whilst participants receiving PIL B were 22% more likely to be recruited, there was no statistically significant effect of PIL type on recruitment.

#### 3.4.2 Secondary Analysis

##### 3.4.2.1 Retention

Of the 88 participants randomised into the RESTORE II trial, 74 (84.1%) were still participating at the T1 (post‐intervention) time point (35/41 (85.4%) PIL A and 39/47 (83.0%) PIL B). The logistic regression gave an OR of 0.84 (95% CI 0.26 to 2.65, *p* = 0.76), meaning that whilst participants who received PIL B were 16% less likely to be retained at T1, there was no statistically significant effect of PIL type on retention.

### 3.5 DMQ

Information pertaining to DMQ response and completion rates is presented in Table 4. The overall DMQ total mean score was 29.17 (SD 4.7). No significant difference in total mean DMQ score was observed between the groups (PIL A 29.19 [SD 4.4] vs. PIL B 29.1 [SD 5.1], mean difference 0.03, 95% CI −1.64 to 1.69, *p* = 0.49). Table 5 summarises the responses to Questions 1–9 on the DMQ scale. Responses to open‐ended questions 10, 11 and 12 are presented in Table 4.

**Table 4:** Decision‐Making Questionnaire (DMQ) findings.

Item	Standard PIL A (*n* = 154)	PPI informed PIL B (*n* = 153)	Overall (*n* = 307)
Total number of DMQs returned, *n* (%)	73 (47.4)	71. (46.4)	144 (46.9)
Number of returned DMQs fully completed, *n* (%)	65 (89.0)	65 (91.5)	130 (90.3)
Number of DMQs returned by host trial consenters, *n* (%)	30 (73.2)	32 (68.1)	62 (70.5)
Number of DMQ returned by non‐consenters to host trial, *n* (%)	43 (38.0)	39 (36.8)	82 (37.4)
Total DMQ score, mean (SD)	29.19 (4.4)	29.1 (5.1)	29.17 (4.7)
Responses to open‐text questions:	PIL A responses (*N*)	PIL B responses (*N*)	Total responses (*N*)
Q10: ‘*Is there any additional information that you would have wanted?’*	10	11	21
Key responses included:			
Seeking information on the timing/dates of intervention and assessments	6	6	12
Reported no further information needed	3	1	4
Seeking clarity on definitions, for example, usual care, current treatment and home‐based exercise	0	2	2
Q11: *‘Identify aspects of information that were explained well’*	37	38	75
Key responses included:			
All of the information	13	17	30
The assessments	6	1	7
The RESTORE II Rehabilitation Programme (Exercise, Diet and Education)	7	10	17
The possible benefits and risks of participation	3	2	5
The individual commitment required for participation	7	1	8
Q12: *‘Do you have any other comments?’*	23	15	38
Key responses included:			
Travel burden prohibited participation	6	7	13
Family commitments prohibited participation	0	3	3
Work commitments prevented participation	1	1	2
Altruism motivated participation	1	1	2
Declined participation as too far into recovery	2	0	2

**Table 5:** DMQ item response.

		Very hard	Hard	Ok	Easy	Very easy	Missing
1. The information I saw about the RESTORE II trial was easy to understand.	PIL A, *n* (%)	0 (0.0)	2 (2.7)	13 (17.8)	32 (43.8)	19 (26.0)	7 (9.6)
	PIL B, *n* (%)	0 (0.0)	1 (1.4)	17 (23.9)	29 (40.9)	18 (25.4)	6 (8.5)
	Overall, *n* (%)	0 (0.0)	3 (2.1)	30 (20.8)	61 (42.4)	37 (25.7)	13 (9.0)
		**Not at all**	**Not really**	**Not sure**	**Yes mostly**	**Yes completely**	**Missing**
2. After seeing the information about the RESTORE II trial, I knew what taking part would be like.	PIL A, *n* (%)	0 (0.0)	1 (1.4)	5 (6.9)	43 (58.9)	17 (23.3)	7 (9.6)
	PIL B, *n* (%)	1 (1.4)	1 (1.4)	3 (4.2)	38 (53.5)	22 (31.0)	6 (8.5)
	Overall, *n* (%)	1 (0.7)	2 (1.4)	8 (5.6)	81 (56.3)	39 (27.1)	13 (9.0)
3. The information helped me understand how my treatment or care might change if I took part in the RESTORE II trial.	PIL A, *n* (%)	0 (0.0)	4 (5.5)	11 (15.1)	30 (41.1)	18 (24.7)	10 (13.7)
	PIL B, *n* (%)	1 (1.4)	2 (2.8)	9 (12.7)	33 (46.5)	20 (28.2)	6 (8.5)
	Overall, *n* (%)	1 (0.7)	6 (4.2)	20 (13.9)	63 (43.8)	38 (26.4)	16 (11.1)
4. The possible benefits of taking part in the RESTORE II trial were made clear in the information.	PIL A, *n* (%)	0 (0.0)	0 (0.0)	1 (1.4)	32 (43.8)	31 (42.5)	9 (12.3)
	PIL B, *n* (%)	0 (0.0)	0 (0.0)	5 (7.0)	31 (43.7)	26 (36.6)	9 (12.7)
	Overall, *n* (%)	0 (0.0)	1 (0.7)	6 (4.2)	63 (43.8)	57 (39.6)	18 (12.5)
5. The possible disadvantages of taking part in the RESTORE II trial were made clear in the information.	PIL A, *n* (%)	0 (0.0)	1 (1.4)	11 (15.1)	24 (32.9)	28 (38.4)	9 (12.3)
	PIL B, *n* (%)	0 (0.0)	0 (0.0)	8 (11.3)	26 (36.6)	27 (38.0)	10 (14.1)
	Overall, *n* (%)	0 (0.0)	1 (0.7)	19 (13.2)	50 (34.7)	55 (38.2)	19 (13.2)
6. The information about the RESTORE II trial helped me discuss the trial with the researcher who asked me to take part.	PIL A, *n* (%)	4 (5.5)	5 (6.9)	11 (15.1)	18 (24.7)	16 (21.9)	19 (26.0)
	PIL B, *n* (%)	6 (8.5)	3 (4.2)	7 (9.9)	18 (25.4)	21 (29.6)	16 (22.5)
	Overall, *n* (%)	10 (6.9)	8 (5.6)	18 (12.5)	36 (23.2)	37 (25.7)	35 (24.3)
7. The information about the RESTORE II trial helped me to discuss taking part with my spouse or family.	PIL A, *n* (%)	2 (2.7)	1 (1.4)	3 (4.1)	23 (31.5)	32 (43.8)	12 (16.4)
	PIL B, *n* (%)	4 (5.6)	1 (1.4)	2 (2.8)	26 (36.6)	31 (43.7)	7 (9.9)
	Overall, *n* (%)	6 (4.2)	2 (1.4)	5 (3.5)	49 (34.0)	63 (43.8)	19 (13.2)
8. I am confident that I have made the right decision about whether or not to take part in the RESTORE II trial.	PIL A, *n* (%)	0 (0.0)	0 (0.0)	2 (2.7)	20 (27.4)	41 (56.2)	10 (13.7)
	PIL B, *n* (%)	0 (0.0)	0 (0.0)	3 (4.2)	19 (26.8)	42 (59.2)	7 (9.9)
	Overall, *n* (%)	0 (0.0)	0 (0.0)	5 (3.5)	39 (27.1)	83 (57.6)	17 (11.8)
9. In all, the information about the RESTORE II trial helped me make my decision about whether or not to take part.	PIL A, *n* (%)	1 (1.4)	2 (2.7)	1 (1.4)	23 (31.5)	35 (48.0)	11 (15.1)
	PIL B, *n* (%)	0 (0.0)	2 (2.8)	2 (2.8)	23 (32.4)	37 (52.1)	7 (9.9)
	Overall, *n* (%)	1 (6.9)	4 (2.8)	3 (2.1)	46 (31.9)	72 (50.0)	18 (12.5)

## 4 Discussion

PPI is essential to ensuring patients are at the heart of clinical research, and increasingly, the efficacy of PPI in trials is being explored by trial methodologists [11]. This SWAT applied PPI purposefully to improve the content, structure and visual appeal of the PIL for the RESTORE II trial. However, the resultant PPI‐informed PIL did not lead to significant improvements in recruitment, retention, or quality of decision‐making for the RESTORE II trial.

An overall recruitment rate of 28.7% was achieved for the RESTORE II trial. Whilst the rate was slightly higher for the SWAT intervention group (PIL B), the difference was not statistically significant. This is akin to the findings of another SWAT by Dwyer et al., which also compared a standard PIL to a PPI informed PIL and the odds ratio between the groups for consent was 0.94 (95% CI −0.62 to 0.49) [23]. Notably, the recruitment rate to our host trial is much lower than the 40.4% achieved by the RESTORE I Pilot RCT conducted between 2016 and 2017 [14]. This lower‐than‐anticipated accrual rate may be attributed to many factors. First, the RESTORE II trial was due to commence in Spring of 2020, but due to restrictions associated with the Covid‐19 pandemic, the trial was delayed and did not commence until Spring of 2022. The pandemic saw an unprecedented increase in the uptake of video calling and a rise in the acceptance of the remote delivery of healthcare via telehealth, including the provision of rehabilitation programmes [24, 25]. As a result, attitudes towards face‐to‐face changed, and patients were much less open to hospital‐based rehabilitation than previously [26, 27, 28]. Due to the nature of the SWAT, the investigators were restricted in their approach to recruitment. To maintain blinding to the SWAT intervention, recruitment was limited to a letter‐based invitation only. It was unforeseen that this lack of face‐to‐face recruitment may have detrimentally impacted the accrual rate. Prior work by this group has highlighted how direct recruitment by healthcare professionals can improve recruitment rates to exercise‐based cancer rehabilitation trials [7]. Furthermore, the post‐pandemic changes in attitude to hospital‐based rehabilitation may also have contributed to the lower retention rate achieved by the RESTORE II trial in comparison to its pilot (93% vs. 83%) [29].

The inclusion of the DMQ in this SWAT provides a better understanding of motivators and barriers to rehabilitation trial participation in cancer survivorship. In the context of cancer survivorship, key motivators for trial participation include wanting access to the best or newest treatments and wanting closer supervision by one's medical team [30, 31]. In addition, high levels of altruism are reported amongst cancer survivors with a resounding sense of needing to give back, and making things better for those in the future, and this was reported in our DMQ responses [32], and corresponds with findings from the pilot RCT of this intervention [33, 34]. Reasons for research non‐participation are often poorly understood, but the DMQ highlighted some key reasons for non‐participation in the RESTORE II trial. The three most frequently reported barriers to participation were travel, family and work commitments, which align strongly with findings from systematic reviews of exercise oncology trials [6, 35] and an umbrella review on research participation [36]. As previously mentioned, the emergence of telehealth as an acceptable mode of rehabilitation delivery has meant that trialists should endeavour to design intervention trials with greater flexibility to overcome potential personal barriers to participation [28], to maximise trial accrual and retention.

Despite the lack of statistically significant findings in this SWAT, it is important to highlight the immense value PPI brings to clinical trials [37]. Over the past few decades, there has been a switch from PPI being a tokenistic gesture in clinical trials to becoming a meaningful part of the trial design and implementation process [12]. Increasingly, its inclusion is expected, if not mandated, by higher education institutions and research funders [38]. The rationale to include PPI in clinical trials is clear: for research to be valid, it must incorporate the voice of patients at its core [39]. We have moved beyond a tokenistic approach, and increasingly, trial methodologists are actively quantifying how PPI can enhance the conduct of clinical trials [40]. Whilst this SWAT did not show a statistically significant effect of PPI on our endpoints of recruitment, retention and quality of decision‐making (DMQ), it is plausible that perhaps a PPI‐informed PIL alone may be insufficient to influence these endpoints. Indeed, the aforementioned work of Dwyer et al. [23] suggested that when all relevant information is presented, the format of presentation may have a limited impact. Therefore, rather than PILs being developed solely by researchers or PPI groups, collaborative approaches are needed to produce evidence‐based PILs which meet readability and regulatory requirements [23]. Such efforts should be integral to a patient‐centred approach to recruitment, retention and decision‐making. For example, Chhatre et al. [41] proposed a conceptual model of patient‐centred recruitment and retention, demonstrating that proactive, multilevel strategies, focusing on the concepts of trust, communication, attitude and expectations, can positively impact trial participation. This highlights that, rather than relying on isolated interventions targeting a single aspect of recruitment or retention, a comprehensive approach is required across the entire process. Finally, while this SWAT focused on recruitment and retention, PPI has also been reported to be effective in assisting researchers to secure funding, developing trial protocols and selecting appropriate trial endpoints [10, 42]. In the future, trial methodologists should continue to investigate how PPI can be best incorporated to enhance aspects of trial conduct.

This SWAT had some strengths and limitations. Key strengths of this SWAT include the use of PPI to inform the development of the interventional PIL, and the randomised double‐blind approach to assessing its effectiveness. However, SWAT embedment does increase trial costs. Additional costs accrued due to the SWAT included the provision of gratuity vouchers, refreshments and parking costs for PPI members, international travel costs of the facilitator, graphic design and printing costs. The lack of diversity in the Phase 1 PPI group is also a limitation. Whilst the PPI group were reflective of the UGI cancer population in the Republic of Ireland (mostly older white males), it was not reflective of other ethnic groups. In line with best practice, future work should aim to be more inclusive. Of note, future work needs to engage with non‐English speakers, those with low health literacy and those that are unfamiliar with the concept of clinical trials. A significant limitation of Phase 2 was the lack of demographic data for non‐consenters to the RESTORE II trial. Due to the General Data Protection Regulations and Health Research Regulations, such data could not be processed without informed consent. Finally, the low return rate of DMQs limited the ability to assess the impact of the interventional PIL on the quality of decision‐making around the trial. Accordingly, future work in this area should investigate strategies to optimise the return rate of DMQs.

## 5 Conclusions

A PPI‐informed PIL did not lead to significant improvements in recruitment, retention, and quality of decision‐making for the RESTORE II Trial. These findings suggest that a PPI‐informed PIL alone may be insufficient to impact these endpoints. Nevertheless, trial methodologists should continue to investigate how PPI engagement can best enhance the conduct of trials through the development and evaluation of multilevel, patient‐centred strategies for recruitment and retention.

## Author Contributions

**Linda O'Neill:** conceptualisation, methodology, investigation, visualisation, formal analysis, project administration, writing – original draft, writing – review and editing. **Peter Knapp:** conceptualisation, methodology, supervision, investigation, writing – review and editing. **Suzanne L. Doyle:** conceptualisation, methodology, funding acquisition, writing – review and editing. **Sanela Begic:** investigation, project administration, writing – review and editing. **Emily Smyth:** investigation, project administration, writing – review and editing. **Neil Kearney:** investigation, project administration, writing – review and editing. **Sophie Grehan:** investigation, project administration, writing – review and editing. **Adwoa Parker:** methodology, writing – review and editing. **Peter Browne:** funding acquisition, writing – review and editing. **Ricardo Segurado:** methodology, formal analysis, writing – review and editing. **Deirdre Connolly:** funding acquisition, writing – review and editing. **Jacintha O'Sullivan:** funding acquisition, writing – review and editing. **John V. Reynolds:** funding acquisition, writing – review and editing. **Emer Guinan:** conceptualisation, methodology, supervision, funding acquisition, writing – review and editing. **Juliette Hussey:** conceptualisation, methodology, supervision, funding acquisition, writing – review and editing.

## Ethics Statement

For Phase I, Ethical Approval was obtained from the Tallaght University Hospital/St James's Hospital Research Ethics Committee (REC:2019‐09 List 35 (08)). Ethical Approval for Phase II was obtained as part of the host trial ethics application to the Tallaght University Hospital/St James's Hospital (REC: 2020‐03 List 11 – Response to Comments (12)) and St Vincent's Hospital Group Ethics Committee (RS19‐066).

## Conflicts of Interest

The authors declare no conflicts of interest.

## Data Availability

The data that support the findings of this study are available upon request from the corresponding author.
